# Association Between Self‐Reported Socio‐Economic Conditions, Air Pollution Sources and Eczema Symptoms Amongst Teenagers in Soshanguve, Tshwane Metropolitan Municipality, Gauteng Province, South Africa

**DOI:** 10.1002/puh2.70325

**Published:** 2026-07-28

**Authors:** Khanyisa Mananga, Mandla Bhuda, Joyce Shirinde

**Affiliations:** ^1^ School of Health Systems and Public Health, Faculty of Health Sciences University of Pretoria Pretoria South Africa; ^2^ Department of Public Health School of Social Sciences University of South Africa Pretoria South Africa

**Keywords:** eczema, environmental tobacco smoke, indoor and outdoor air pollution, socio‐economic factors, South Africa

## Abstract

**Background:**

This study aimed to investigate the association between self‐reported socio‐economic conditions, air pollution and the prevalence of atopic eczema among teenagers in Soshanguve, Tshwane Metropolitan Municipality, Gauteng Province, South Africa.

**Materials and Methods:**

A cross‐sectional study of 2885 schoolchildren aged 13–14 years from 13 high schools in Soshanguve was conducted. Participants completed a validated, modified ISAAC III questionnaire, and data were analysed using univariate and multiple logistic regression in Stata 18 to identify predictors of eczema.

**Results:**

Prevalence estimates indicated that 26.8% of participants self‐reported current eczema symptoms (ES), whereas 23.3% self‐reported eczema ever (EE). Females were more likely to report current ES (adjusted odds ratio [AOR] 2.07) and EE (AOR 1.63). Monthly paracetamol (Panadol) use was strongly associated with self‐reported current ES and EE (AOR 2.37). Environmental and household factors increased risk: Frequent truck traffic near homes elevated the self‐reported current ES (AOR 1.26) and EE (AOR 1.24); cat ownership increased self‐reported current ES (AOR 1.49); dog ownership increased self‐reported EE (AOR 1.39); and exposure to environmental tobacco smoke (ETS) at home for 16–20 days per month raised the likelihood of self‐reported EE (AOR 1.96; *p *< 0.05).

**Conclusion:**

We observed that self‐reported pet ownership, frequent paracetamol use, exposure to ETS and nearby truck traffic were associated with increased odds of eczema among teenagers in Soshanguve.

## Graphical abstract

Self‐reported socio‐economic conditions and air pollution exposures, particularly environmental tobacco smoke and traffic‐related pollution, were associated with increased odds of eczema symptoms among adolescents in Soshanguve, South Africa, highlighting the need for targeted public health interventions.

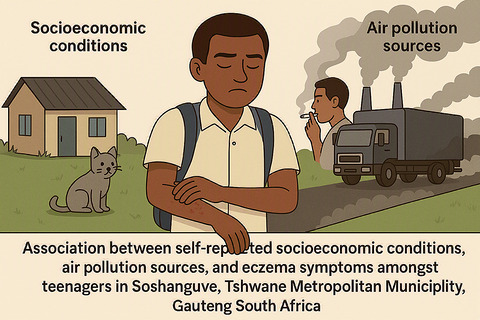

## 1 Background

Air pollution is a significant global health burden, particularly in low‐ and middle‐income countries, which experience the highest levels of exposure [1]. According to the World Health Organization (WHO), 99% of the global population lives in areas where air quality exceeds guideline limits, with elevated pollutant levels [1]. In Africa, South Africa ranks fourth in deaths attributed to air pollution [2]. Cities and townships in South Africa with high carbon emissions, mainly from automobiles, factories and mines, expose residents to air pollution and an increased risk of related diseases [3, 4, 5, 6]. Air pollution has been consistently linked to cardiovascular and respiratory diseases, childhood deaths, hospitalizations, school absenteeism and reduced quality of life [1, 7, 8, 9, 10]. In LMIC's, rising mortality rates have also been associated with air pollution [11]. Studies in major South African cities show a positive association between air pollution exposure and mortality, with children being particularly vulnerable [12, 13]. Evidence suggests that increasing levels of air pollution, which are linked to climate change, will continue to impact human health [14].

Socio‐economic factors (household quality, healthcare access, pet ownership and place of residence) and air pollution (fossil fuels, tobacco smoke and vehicle emissions) influence the development and exacerbation of eczema [15, 16, 17, 18]. Exposure to air pollution sources predominantly occurs via inhalation [1]. Exposure to pollutants, such as nitrogen dioxide, particulate matter and carbon monoxide, has been associated with eczema in children, highlighting the adverse effects of ambient air pollution on skin health [19]. Eczema, or atopic dermatitis, is an inflammatory skin condition characterized by dryness and dysfunction of the outer epidermal layer [20]. Globally, an estimated 223 million people were living with atopic dermatitis in 2022 [20]. The condition contributes to school absenteeism (5.25%) and sleep disturbance (24.1%) [21]. There are limited data on the prevalence, severity, treatment needs and environmental determinants of atopic dermatitis across different geographical regions [20].

Few studies in South Africa have examined eczema in relation to socio‐economic factors in township settings [22, 23, 24, 25]. This study aligns with SDG 3 (Good Health and Well‐Being) and SDG 11 (Sustainable Cities and Communities) [26], addressing a critical knowledge gap as the first to investigate socio‐economic conditions and air pollution sources among high school students in Soshanguve. Although some research has focused on preschool children and air pollution in priority regions such as the Highveld, Bojanala and Waterberg, township settings remain understudied [22, 23, 24]. This study aimed to investigate the association between socio‐economic conditions, air pollution and the prevalence of atopic eczema among teenagers in Soshanguve, Tshwane Metropolitan Municipality, Gauteng Province, South Africa.

## 2 Materials and Methods

### 2.1 Study Setting

The study was conducted in Soshanguve township, located within the City of Tshwane Metropolitan Municipality (CTMM) in Gauteng, South Africa, approximately 30 km north of Tshwane. According to the World Population Review, the population of Soshanguve was estimated at 905,868 in 2024 [4], whereas the 2011 national census reported a population of 403,162 [27]. Soshanguve is a multicultural township with a diverse linguistic profile, the most commonly spoken languages being Sepedi, followed by Setswana, isiZulu, Xitsonga, Afrikaans, English, Siswati and isiNdebele [22]. Approximately 16.5% of the population has no source of income [22].

### 2.2 Study Design, Population and Sampling

The study used a quantitative cross‐sectional design following the ISAAC III protocol (Supporting Information 1) [23, 28] among children aged 13–14 years attending schools in Soshanguve. Data were collected from January to February 2024. Fourteen schools were approached, 13 consented and were purposively selected from the Gauteng Department of Basic Education list; one school was used for a pilot study. Principals approved participation, and consent forms were sent to parents with assent forms for students. Written parental consent and student assent were obtained, resulting in 2885 participants from 3267 eligible students.

### 2.3 Health Outcomes

Self‐reported current eczema symptoms (ES) and eczema ever (EE) were assessed on the basis of participant responses to the questionnaire [22, 23].

**Self‐reported current ES**: Participants were classified as having ES if they answered ‘Yes’ to all three of the following questions:

- Have you ever had an itch that comes and goes over the past 6 months? (Yes/No)
- Have you had this itchy rash at any time in the past 12 months? (Yes/No)
- Has this itchy rash affected any of the following areas: the folds of the elbows, behind the knees, in front of the ankles, under the buttocks, or around the neck, ears or eyes? (Yes/No)

**Self‐reported EE**: Participants were classified as having EE based on ‘Yes’ responses to the following questions:

- Have you ever had eczema? (Yes/No)
- Was the eczema diagnosed by a doctor? (Yes/No)

### 2.4 Socio‐Economic Factors, Air Pollution Sources and Confounders

Self‐reported socio‐economic factors were assessed using dwelling type (brick, mud, corrugated iron and mixed), pet ownership, proximity to healthcare (clinic or hospital) and lifestyle factors such as paracetamol (Panadol) use. Air pollution sources included exposure to environmental tobacco smoke (ETS) at home, school, in transport and in restaurants over the past 30 days (yes/no), maternal smoking (yes/no) and smoking by other household members (yes/no). Participants also reported truck traffic frequency near their residence (never, seldom, frequently and almost all day). This was a self‐reported exposure measure rather than an objective measure based on traffic counts or direct monitoring. Potential confounders included sex, hours spent watching television, mode of transport to school, birthplace (Soshanguve or Mabopane) and school attendance.

### 2.5 Statistical Analysis

Questionnaire data were entered into EpiData and analysed using Stata version 18 (StataCorp, College Station, TX, USA). Descriptive statistics were used to summarize participant characteristics, eczema outcomes, socio‐economic conditions, air pollution sources and potential confounders. Prevalence estimates were calculated using available (non‐missing) responses, with ‘do not know’, ‘not stated’ and ‘other’ responses coded as missing and excluded from the analyses. Missing data were handled using complete case (listwise deletion), resulting in sample sizes ranging from 2702 to 2885 participants across the regression analyses, depending on the variables included in each model. Pearson's chi‐square tests were used to assess associations between categorical variables. Univariable logistic regression was performed to estimate crude (unadjusted) odds ratios (ORs) and 95% confidence intervals (CIs). Variables with *p* < 0.20 in the univariable analyses were included in multiple logistic regression analysis (MLRA) models to estimate adjusted odds ratios (AORs) and 95% CIs. Statistical significance was set at *p* < 0.05.

## 3 Results

Table 1 summarizes the socio‐demographic characteristics of the 2885 participants. Nearly half were male (48.9%) and 46.4% female, with 4.7% missing gender data. Most were aged 12–14 years (95.5%) and 4.5% were 15 years or older. Residential stability was high, with 76.2% living in Soshanguve for 3 years or more, 8.4% for 1–2 years, 5.9% for 6–12 months and 6.6% for less than 6 months. Housing was mainly brick (72.2%), followed by corrugated iron (9.3%), mixed structures (8.5%), mud houses (2.5%) and other types (5.3%).

**TABLE 1:** Socio‐demographic characteristics of study participants (*n* = 2885).

Variable	Total	Percentage
**Gender**		
Male	1410	48.9
Female	1340	46.4
Missing	135	4.7
**Age group (years)**		
12–14	2756	95.5
≥15	129	4.5
**Time lived in area**		
<6 months	189	6.6
6–12 months	171	5.9
1–2 years	242	8.4
≥3 years	2197	76.2
Missing	86	3.0
**Type of house live in**		
Brick	2083	72.2
Mud	72	2.5
Corrugated iron	267	9.3
Combination	246	8.5
Other	152	5.3
Missing	65	2.3
**Use of paracetamol**		
Never	564	19.5
At least once a year	959	33.2
At least once per month	1258	43.6
Missing	104	3.6
**Mode of transport to school**		
Walk	1598	55.4
Taxi/Bus	684	23.7
Motor car	302	10.5
Combination	191	6.6
Other	70	2.4
Missing	40	1.4
**School attendance**		
Never or occasionally	1706	59.1
Once or twice per week	806	27.9
Three or more times per week	281	9.8
Missing	92	3.2
**Distance to nearest clinic or hospital**		
15 min walk/5 min drive	1248	43.3
1 h walk/15 min drive	869	30.1
>1 h walk/>30 min drive	679	23.5
Missing	89	3.1
**Cat at home**		
Yes	252	8.7
No	2603	90.2
Missing	30	1.0
**Dog at home**		
Yes	1111	38.5
No	1738	60.2
Missing	36	1.2

Regarding paracetamol use, 43.6% used it at least monthly, 33.2% at least yearly and 19.5% never. Walking was the main mode of transport to school (55.4%), followed by taxis/buses (23.7%), motor vehicles (10.5%) and mixed modes (6.6%). School attendance was generally good, with 59.1% never or occasionally absent, 27.9% absent once or twice per week and 9.8% absent three or more times per week. Access to health services was adequate: 43.3% lived within a 15‐min walk or 5‐min drive to a clinic or hospital, 30.1% within 1 h or 15‐min drive and 23.5% further away. Pet ownership was low, with 8.7% reporting a cat and 38.5% a dog.

Table 2 summarizes participants’ exposure to self‐reported air pollution sources. Most participants had never smoked (75.0%), and 82.2% were not current smokers. Occasional smoking was reported by 18.3%, whereas 3.7% smoked daily. Among current smokers, 13.0% smoked less than daily and 3.0% smoked daily. Parental smoking differed by gender, with 13.0% reporting maternal smoking and 34.1% reporting paternal smoking. Indoor exposure was common, as 46.4% lived in households with indoor smoking. Passive ETS exposure varied by setting: At home, 19.5% were exposed for 1–6 days and 10.8% for more than 20 days; at school, 5.2% reported exposure for more than 20 days, whereas 64.0% reported none; in transport, 3.5% reported exposure for more than 20 days and 66.0% none; and restaurant exposure for more than 20 days was reported by 4.4%. Environmental exposure was further indicated by frequent truck traffic near homes (37.1%).

**TABLE 2:** Self‐reported air pollution sources of the study participants (*n* = 2885).

Variable	Total	Percentage
**In the past, smoking tobacco**		
Not at all	2163	75.0
Less than daily	529	18.3
Daily	107	3.7
Missing	86	3.0
**Currently smoke tobacco**		
Not at all	2371	82.2
Less than daily	376	13.0
Daily	87	3.0
Missing	51	1.8
**Mother/female guardian smokes cigarettes**		
Yes	374	13.0
No	2511	87.0
**Father/male guardian smokes cigarettes**		
Yes	984	34.1
No	1901	65.9
**Number of people who smoke in the same house as participant**		
Zero	1518	52.6
One or more	1339	46.4
Missing	28	1.0
**Smoking exposure at home (days per month)**		
Never	1682	58.3
1–6 days	564	19.5
7–10 days	117	4.1
16–20 days	55	1.9
More than 20 days	311	10.8
Missing	156	5.4
**Smoking exposure at school (days per month)**		
Never	1845	64.0
1–6 days	441	15.3
7–10 days	98	3.4
16–20 days	44	1.5
More than 20 days	149	5.2
Missing	308	10.7
**Smoking exposure in car/transport (days per month)**		
Never	1903	66.0
1–6 days	374	13.0
7–10 days	99	3.4
16–20 days	44	1.5
More than 20 days	100	3.5
Missing	365	12.7
**Smoking exposure at restaurants (days per month)**		
Never	1872	64.9
1–6 days	338	11.7
7–10 days	98	3.4
16–20 days	59	2.0
More than 20 days	128	4.4
Missing	390	13.5
**Truck traffic near home**		
Never	545	18.9
Seldom	677	23.5
Frequently throughout the day	533	18.5
Almost the whole day	1071	37.1
Missing	59	2.0

Table 3 presents the self‐reported health outcomes for current ES and EE. Overall, 39.5% of participants reported an itchy rash that came and went during the previous 6 months. Among these participants, 78.5% reported experiencing the rash during the past 12 months, and 86.3% indicated that it affected typical flexural areas, including the elbows, knees, ankles, buttocks, neck, ears or eyes. Participants who answered ‘yes’ to all three questions were classified as having current ES, resulting in an overall prevalence of 26.8% (*n* = 773). Overall, 23.3% of participants reported EE, of whom 44.3% indicated that the condition had been diagnosed by a doctor.

**TABLE 3:** Self‐reported health outcomes of the study participants (*n* = 2885).

Health outcomes	Total	Percentage
**Ever itchy rash in the past 6 months**		
Yes	1141	39.5
No	1744	60.5
**Itchy rash at any time in past 12 months**		
Yes	896	78.5
No	245	21.5
**Itchy rash affected place**		
Yes	773	86.3
No	123	13.7
**Age of itchy‐rash first occurrence**		
Age 2–4	98	10.9
Age 5 or more	273	30.5
Can not remember	525	58.6
**Has this rash cleared completely**		
Yes	583	65.1
No	313	34.9
**Nights awake due to itchy rash**		
Never in the past 12 months	239	26.7
Less than one night per week	345	38.5
One or more nights per week	312	34.8
**Current eczema symptoms**		
Yes	773	26.8
No	2112	73.2
**Eczema ever**		
Yes	673	23.3
No	2212	76.7

Table S1 presents the prevalence of current ES and EE by sex among participants with complete information on sex and eczema outcomes. The prevalence of both current EE and ES was higher among females than males (29.0% vs. 19.4% and 35.5% vs. 19.4%, respectively). The overall prevalence estimates reported in Table S1 (24.0% for EE and 27.2% for current ES) differed slightly from those reported in Table 3 because participants with missing information on sex and/or eczema outcomes were excluded from the sex‐stratified analyses.

Table 4 summarizes the results of the MLRA for predictors of current ES. Females had significantly higher odds than males (AOR 2.07, 95% CI: 1.74–2.48). Paracetamol use showed a dose–response pattern, with increased odds among children using it at least once per year (AOR 1.92, 95% CI: 1.42–3.57) and the highest odds among monthly users (AOR 2.37, 95% CI: 1.86–3.17). Exposure to truck traffic was a significant predictor (AOR 1.26, 95% CI: 1.05–1.51), as was cat ownership (AOR 1.49, 95% CI: 1.09–2.02). ETS exposure was also associated with current ES, including smoking at home for more than 20 days (AOR 1.60, 95% CI: 1.21–2.12) and exposure in restaurants for 7–10 days (AOR 1.72, 95% CI: 1.18–2.67). Living in a corrugated iron house showed increased odds but was not supported as an independent predictor.

**TABLE 4:** Socio‐economic conditions and air pollution sources predictors of current eczema symptoms.

Variable	Unadjusted OR (95% CI)	*p* value	Adjusted OR (95% CI)	*p* value
**Gender**				
Male	1	—	1	—
Female	2.28 (1.92–2.73)	0.000	2.07 (1.74–2.48)	**0.000**
**Age group (years)**				
12–14	1	—	—	—
≥15	0.71 (0.48–1.09)	0.149	—	—
**Time lived in area**				
<6 months	1	—	—	—
6–12 months	0.95 (0.53–1.57)	0.871	—	—
1–2 years	1.20 (0.74–1.81)	0.431	—	—
≥3 years	1.48 (0.84–1.67)	0.031	—	—
**Type of house children live in**				
Brick	1	—	—	—
Mud	0.86 (0.43–1.50)	0.686	—	—
Corrugated iron	1.34 (1.03–1.76)	0.030	—	—
Combination	1.07 (0.79–1.43)	0.654	—	—
Other	0.92 (0.61–1.37)	0.723	—	—
**Use of paracetamol**				
Never	1	—	1	—
Once a year	2.19 (1.66–2.88)	0.000	1.92 (1.42–3.57)	**0.000**
Once per month	2.95 (2.26–3.89)	0.000	2.37 (1.86–3.17)	**0.000**
**Mode of transport to school**				
Walk	1	—	—	—
Taxi/Bus	0.93 (0.73–1.14)	0.434	—	—
Motor car	0.89 (0.67–1.19)	0.415	—	—
**School attendance**				
Never/occasionally	1	—	—	—
1–2/week	1.16 (0.95–1.42)	0.114	—	—
≥3/week	1.24 (0.94–1.69)	0.118	—	—
**Distance to nearest clinic or hospital**				
15 min walk/5 min drive	1	—	—	—
1 h walk/15 min drive	1.08 (0.89–1.37)	0.446	—	—
>1 h walk/>30 min drive	1.01 (0.82–1.25)	0.924	—	—
**Trucks pass on the street**				
Never/seldom	1	—	1	—
Frequently/whole day	1.33 (1.11–1.57)	0.001	1.26 (1.05–1.51)	**0.012**
**Pets at home**				
Cat (Yes)	1.40 (1.06–1.85)	0.017	1.49 (1.09–2.02)	**0.010**
Dog (Yes)	1.15 (0.97–1.37)	0.017	—	—
**Past smoking in household**				
Not at all	1	—	—	—
Less than daily	1.17 (0.95–1.44)	0.176	—	—
Daily	0.71 (0.44–1.15)	0.168	—	—
**Current smoking**				
Not at all	1	—	—	—
Less than daily	1.31 (1.03–1.67)	0.026	—	—
Daily	1.62 (0.45–3.10)	0.223	—	—
**Mother/female guardian smokes**				
No	1	—	—	—
Yes	1.23 (0.92–1.35)	0.085	—	—
**Father/male guardian smokes**				
No	1	—	—	—
Yes	1.21 (0.96–1.32)	0.025	—	—
**People smoking in the house**				
Zero	1	—	—	—
One or more	0.99 (0.84–1.17)	0.953	—	—
**Smoking exposure at home**				
Never	1	—	1	—
1–6 days	1.17 (1.14–1.71)	0.141	1.17 (0.93–1.47)	0.165
7–10 days	1.03 (0.92–2.21)	0.824	1.14 (0.73–2.21)	0.546
16–20 days	1.97 (0.46–1.43)	0.985	1.20 (0.64–1.36)	0.575
>20 days	1.36 (1.04–1.79)	0.003	1.60 (1.21–2.12)	**0.001**
**Smoking exposure at restaurant**				
Never	1	—	1	—
1–6 days	1.16 (0.98–1.59)	0.264	1.16 (0.89–1.51)	0.254
7–10 days	1.65 (1.08–2.54)	0.020	1.72 (1.18–2.67)	**0.014**
16–20 days	0.73 (0.79–2.57)	0.333	0.70 (0.79–2.57)	0.291
>20 days	1.89 (0.81–1.87)	0.001	1.71 (1.17–2.57)	**0.005**

*Note:* Variables with *p* < 0.20 in the univariable analyses were included in the multiple logistic regression model. Adjusted estimates with *p* < 0.05 are shown in bold. Complete case analysis was used to handle missing data; therefore, the number of observations varied across regression models depending on the variables included.

Abbreviations: AOR, adjusted odds ratio; CI confidence interval; OR, odds ratio.

Table 5 summarizes the results of the MLRA for predictors of EE. Female participants had significantly higher odds of EE than males (AOR 1.63, 95% CI: 1.34–1.93). Frequent paracetamol use was also associated with increased odds of EE, with monthly use showing the strongest association (AOR 2.37, 95% CI: 1.36–2.17). Environmental exposures were important predictors, including frequent truck traffic near the home (AOR 1.24, 95% CI: 1.03–1.49), dog ownership (AOR 1.39, 95% CI: 1.15–1.69) and ETS exposure at home for 16–20 days per month (AOR 1.96, 95% CI: 0.44–1.36).

**TABLE 5:** Socio‐economic conditions and air pollution sources predictors of ever eczema.

Variable	Unadjusted OR (95% CI)	*p* value	Adjusted OR (95% CI)	*p* value
**Gender**				
Male	1	—	1	—
Female	1.69 (1.24–1.73)	0.000	1.63 (1.34–1.93)	**0.000**
**Age group (years)**				
12–14	1	—	—	—
≥15	1.09 (0.68–1.56)	0.849	—	—
**Time lived in area**				
<6 months	1	—	—	—
6–12 months	1.29 (0.73–2.17)	0.309	—	—
1–2 years	1.14 (0.74–1.81)	0.516	—	—
≥3 years	1.16 (0.84–1.67)	0.443	—	—
**Type of house**				
Brick	1	—	—	—
Mud	1.02 (0.53–1.75)	0.986	—	—
Corrugated iron	1.24 (0.93–1.26)	0.151	—	—
Combination	0.95 (0.99–1.88)	0.750	—	—
Other	0.92 (0.61–1.37)	0.622	—	—
**Use of paracetamol**				
Never	1	—	1	—
≥1×/year	1.76 (1.32–2.43)	0.000	1.66 (1.22–1.87)	**0.001**
≥1×/month	2.52 (1.56–3.29)	0.000	2.37 (1.36–2.17)	**0.000**
**Transport to school**				
Walk	1	—	—	—
Taxi/Bus	0.99 (0.83–1.23)	0.934	—	—
Motor car	1.16 (0.81–1.37)	0.215	—	—
Combination	1.50 (1.07–2.01)	0.016	—	—
Other	0.49 (0.27–1.34)	0.052	—	—
**Absence from school**				
Never/occasionally	1	—	—	—
1–2×/week	1.16 (0.95–1.42)	0.131	—	—
≥3×/week	1.27 (0.94–1.69)	0.108	—	—
**Distance to clinic**				
15 min walk/5 min drive	1	—	—	—
1 h walk/15 min drive	1.12 (0.92–1.37)	0.246	—	—
>1 h walk/>30 min drive	0.96 (0.74–1.20)	0.724	—	—
**Truck traffic near home**				
Never/seldom	1	—	1	—
Frequently/whole day	1.32 (0.72–1.45)	0.002	1.24 (1.03–1.49)	**0.002**
**Pets at home**				
Cat (Yes)	1.31 (0.98–1.75)	0.064	—	—
Dog (Yes)	1.24 (1.04–1.48)	0.017	1.39 (1.15–1.69)	**0.017**
**Past smoking in household**				
Not at all	1	—	—	—
Less than daily	1.22 (0.98–1.51)	0.076	—	—
Daily	0.94 (0.62–1.57)	0.968	—	—
**Current smoking**				
Not at all	1	—	1	—
Less than daily	1.31 (1.02–1.67)	0.030	1.29 (1.02–1.67)	0.046
Daily	1.72 (1.45–3.10)	0.023	0.82 (0.85–3.10)	**0.023**
**Mother smokes**				
No	1	—	—	—
Yes	1.15 (0.92–1.35)	0.254	—	—
**Father smokes**				
No	1	—	—	—
Yes	1.13 (0.96–1.32)	0.180	—	—
**People smoking in house**				
Zero	1	—	—	—
One or more	0.98 (0.82–1.17)	0.853	—	—
**Smoking exposure at home**				
Never	1	—	1	—
1–6 days	0.97 (1.14–1.71)	0.905	0.95 (1.06–1.66)	**0.010**
7–10 days	1.03 (0.92–2.21)	0.867	1.00 (0.89–2.21)	0.176
16–20 days	1.97 (0.46–1.43)	0.017	1.96 (0.44–1.36)	**0.019**
>20 days	1.36 (1.04–1.79)	0.025	1.36 (1.12–2.07)	**0.026**
**Smoking exposure at school**				
Never	1	—	—	—
1–6 days	1.15 (0.90–1.46)	0.253	—	—
7–10 days	1.12 (0.71–1.87)	0.696	—	—
16–20 days	0.77 (0.35–1.60)	0.592	—	—
>20 days	1.22 (0.87–1.79)	0.268	—	—
**Smoking exposure in vehicle**				
Never	1	—	—	—
1–6 days	1.21 (0.94–1.56)	0.156	—	—
7–10 days	0.88 (0.53–1.45)	0.670	—	—
16–20 days	1.79 (0.94–3.34)	0.074	—	—
>20 days	1.26 (0.72–1.87)	0.534	—	—
**Smoking exposure at restaurants**				
Never	1	—	—	—
1–6 days	1.28 (0.98–1.69)	0.064	—	—
7–10 days	1.01 (0.63–1.64)	0.953	—	—
16–20 days	1.41 (0.79–2.57)	0.285	—	—
>20 days	1.21 (0.81–1.87)	0.383	—	—

*Note:* Variables with *p* < 0.20 in the univariable analyses were included in the multiple logistic regression model. Adjusted estimates with *p* < 0.05 are shown in bold. Complete case analysis was used to handle missing data; therefore, the number of observations varied across regression models depending on the variables included.

Abbreviations: AOR, adjusted odds ratio; CI, confidence interval; OR, odds ratio.

## 4 Discussion

This study investigated the association between self‐reported socio‐economic conditions, air pollution sources and the self‐reported prevalence of eczema among teenagers in Soshanguve Township, Tshwane Metropolitan Municipality. The overall prevalence of self‐reported current ES was 26.8%, whereas the prevalence of EE was 23.3% (*n* = 2885). These rates were notably higher than those reported in a recent 2024 study conducted in Soshanguve and Mabopane among preschool children, which reported current ES and EE prevalences of 13.3% and 11.9%, respectively [23]. The difference may reflect age‐related changes in symptom recognition, environmental exposures and heightened reporting accuracy among older children [29]. Although eczema is not a life‐threatening condition, it may result in skin damage and is associated with an increased risk of sleep disorders, depression, anxiety, fatigue, lack of attention and impulsivity among adolescents [30], which adversely affect their quality of life and psychosocial well‐being [31].

A significant gender variation was observed in the current study. Female participants had higher odds of reporting eczema than males, with an AOR of 1.63 for EE and 2.07 for ES. This contrasts with findings from the preschool study conducted in the same region, where males showed a slightly higher prevalence [23]. These variations may be influenced by biological differences, such as hormonal changes during adolescence and differences in skin barrier function [32]. A study from England similarly reported fluctuating eczema prevalence across the lifespan, with higher rates in male infants and increased prevalence in females during adolescence [33].

Living in corrugated iron structures showed higher crude odds of ES, but the association was not significant after adjustment. Studies in other South African settings, such as Polokwane, have reported higher eczema prevalence among children in informal housing and rural areas [24]. Similarly, a study in Soshanguve among preschool children found an association between eczema and housing materials such as corrugated iron, brick and mud [22]. These mixed findings indicate that housing conditions may interact with other environmental and socio‐economic variables to influence eczema risk [34].

Owning a pet, particularly cats, was associated with an increased risk of ES in this study. Several international studies have reported similar associations, suggesting that pet dander and indoor allergens may trigger or worsen atopic dermatitis in sensitized children [35]. Medication use, specifically paracetamol, also showed a strong dose‐dependent association. Children who used paracetamol at least once per year had significantly higher odds of current ES, and those using it monthly had more than double the odds. Previous research has similarly shown that paracetamol use within the past 12 months is associated with an increased risk of eczema and other atopic conditions [36]. Although the underlying mechanism remains under investigation, oxidative stress and depletion of glutathione have been proposed as contributing factors.

Prolonged exposure to ETS at home (more than 20 days) was significantly associated with an increased risk of developing ES Children spend a substantial proportion of their time indoors (approximately 80%), and living with a smoking parent or caregiver therefore increases sustained exposure to harmful indoor pollutants [37]. Evidence from South Africa shows a consistent pattern across different settings and age groups [23, 38, 39]. A study conducted in the Ekurhuleni Municipality among schoolchildren aged 12–14 years also reported a significant association between ETS exposure and ES [23]. Similarly, a more recent study conducted in Barberton (2021–2023) among children aged 6–8 years demonstrated a significant association between ETS exposure with ES and EE [25]. These findings suggest that, despite existing tobacco control legislation in South Africa, children continue to experience substantial exposure to ETS in home environments [23]. Mechanistically, ETS is a major contributor to indoor fine particulate matter (PM_2.5_), which has been increasingly implicated in the development and exacerbation of atopic dermatitis through pathways involving epidermal barrier dysfunction, immune dysregulation, oxidative stress and enhanced allergic sensitisation [40].

Children living in areas where trucks frequently passed throughout the day had significantly increased odds of current ES. These results align with a 2023 Lagos study, which found a higher prevalence of eczema among children exposed to heavy truck traffic near residential areas [41]. Although truck traffic exposure in our study was based on participants' self‐reported estimates rather than objective traffic counts or direct traffic measurements, the observed association is biologically plausible. Truck emissions contribute to elevated levels of particulate matter, and nitrogen oxides pollutants known to exacerbate inflammatory skin conditions [42]. For example ES are reported to increase by 10.28% (95% CI: 3.24–17.79) per 10 ppb increase in NO_2_ [43]. Therefore, the CTMM should strengthen the implementation of diesel vehicle emission control measures, including routine inspection and maintenance programmes. In addition, the CTMM should consider reviewing its air quality management plan to enhance strategies aimed at reducing exposure to air pollution sources, particularly in residential areas and townships [44, 45, 46]. For example, a recent health risk assessment conducted in Mabopane, in close proximity to Soshanguve (approximately 4 km away), reported PM_2.5_ concentrations of approximately 10.4 µg/m^3^, exceeding the WHO guideline value of 5 µg/m^3^ [43]. The study also identified trace elements, including nickel, which may pose additional health risks [47].

Several limitations should be noted. First, the cross‐sectional design limits causal inference as exposures and outcomes were measured simultaneously. Second, data were self‐reported, relying on participants’ recall, which may lead to under or over‐reporting of eczema. Third, although several confounders such as gender (sex) and transportation were adjusted for, other important factors, such as genetic predisposition, detailed environmental exposures, humidity, temperature and healthcare access, were not assessed. Fourth, truck traffic exposure was based on self‐reported frequency rather than objective traffic measurements, potentially resulting in exposure misclassification. Strengths include the use of a validated questionnaire ISAAC and a large sample size. Future studies should therefore incorporate objective exposure assessments (e.g., air quality monitoring, accurate vehicle counts, dietary intake measures and green space exposure) and longitudinal study designs to better clarify causal pathways.

## 5 Conclusion

We observed that self‐reported pet ownership, frequent paracetamol use, exposure to ETS and nearby truck traffic were associated with increased odds of eczema among teenagers in Soshanguve. These findings highlight the importance of environmental and household exposures associated with eczema among adolescents in underserved communities in South Africa. Public health measures aimed at reducing exposure to ETS and traffic‐related air pollution, together with improved awareness of eczema risk factors and early detection and management, may help reduce the burden of eczema. Longitudinal studies are warranted to confirm these associations and clarify potential causal pathways.

## Author Contributions

Khanyisa Mananga contributed to the conceptualization of the study, data curation, formal analysis, methodology development, validation, visualization and writing of the original draft of the manuscript. Mandla Bhuda contributed to the conceptualization, data curation, formal analysis, methodology, validation and visualization and was responsible for writing – review and editing, supervision, investigation and software. Joyce Shirinde contributed to the conceptualization, data curation, formal analysis, methodology, validation and visualization and was responsible for writing the original draft, writing – review and editing, supervision, investigation, software and funding acquisition. All authors have read and approved the final manuscript.

## Funding

This study was supported through grants linked to the supervisors from the National Research Foundation (NRF) and the South African Medical Research Council (SAMRC).

## Ethics Statement

This study was approved by the Research Ethics Committee of the Faculty of Health Sciences, University of Pretoria (Ethics Numbers 666/2024). Additional approval was obtained from the Gauteng Department of Basic Education and the Tshwane North District.

## Consent

The study adhered to ethical research principles, with informed consent obtained from parents and assent from participating children before data collection. Data from the participants were kept confidential.

## Conflicts of Interest

The authors declare no conflicts of interest.

## Supporting information




**Supporting information file 1:** puh270325‐sup‐0001‐SuppMat.docx

**Supporting information file 2:** puh270325‐sup‐0002‐TableS1.docx

## Data Availability

The datasets analysed in this study are available from the corresponding author upon reasonable request.
