# Supplementary material for: Association Between Self‐Reported Socio‐Economic Conditions, Air Pollution Sources and Eczema Symptoms Amongst Teenagers in Soshanguve, Tshwane Metropolitan Municipality, Gauteng Province, South Africa
Source: Public Health Chall. 2026 Jul 28;5(3):e70325. doi: 10.1002/puh2.70325 (PMC13411282; doi:10.1002/puh2.70325)
Supplement: Supplementary file 1 — Supporting information file 1: puh270325‐sup‐0001‐SuppMat.docx [file PUH2-5-e70325-s001.docx]

# **Supplementary 1:** **ISAAC Questionnaire**

**Instructions for completing the questionnaire**

Examples of instructions for completing questionnaire and demographic questions are given below.

On this sheet are questions about your child’s name, school, and birth dates. Please write your answers to these questions in the space provided.

All other questions require you to tick your answer in a box. If you make a mistake, put a cross in the box and tick the correct answer. Tick only one option unless otherwise instructed.

134-14

Examples of how to mark questionnaires: Age years

To answer Yes/No, put a cross in the appropriate box as per example below:

| YES |  |
| --- | --- |
| NO | **X** |

----------------------------------------------------------------------------------------------------------------------------------------------------------

**SUBURB/ TOWNSHIP/SECTION**

**WHERE YOU LIVES**:

**SCHOOL**:

**TODAY’S DATE**:

**YOUR NAME**: (code sheet will be used to

Record the names separately – names will not

for be used reporting)

**YOUR AGE**: years

**YOUR WEIGHT**? kg

**YOUR HEIGHT?**  Meters /centimeters (Please circle the measurement you used)

**(Tick all your answers for the rest of the questionnaire)**

**Are you a**: **MALE**  **FEMALE
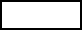
 OTHER**

**Core questionnaire for asthma**

1. Have you ever had wheezing or whistling in the chest at any time in the past?

| YES |  |
| --- | --- |
| NO |  |

**IF YOU HAVE ANSWERED “NO” PLEASE SKIP TO QUESTION 6**

1. Have you had wheezing or whistling in the chest in the past 12 months?

| YES |  |
| --- | --- |
| NO |  |

**IF YOU HAVE ANSWERED “NO” PLEASE SKIP TO QUESTION 6**

1. How many attacks of wheezing have you had in the past 12 months?

| NONE |  |
| --- | --- |
| 1 TO 3 |  |
| 4-14 |  |
| More than twelve |  |

1. In the past 12 months, how often, on average, has your sleep been disturbed due to wheezing?

| Never woken with wheezing |  |
| --- | --- |
| Less than one night per week |  |
| One or more nights per week |  |

1. In the past 12 months, has wheezing ever been severe enough to limit your speech to only one or two words at a time between breaths?

| YES |  |
| --- | --- |
| NO |  |

1. Have you ever had asthma?

| YES |  |
| --- | --- |
| NO |  |

1. Was the asthma diagnosed by a medical doctor or nurse?

| YES |  |
| --- | --- |
| NO |  |

1. In the past 12 months, has your chest ever sounded wheezy during or after playing?

| YES |  |
| --- | --- |
| NO |  |

1. In the past 12 months, have you had a dry cough at night, apart from a cough associated with a cold or chest infection?

| YES |  |
| --- | --- |
| NO |  |

**Core questionnaire for rhinitis- (when a reaction occurs that causes nasal congestion, runny nose, sneezing and itching)**

1. Have you ever had a problem with sneezing, or a runny, or blocked nose when you DID NOT have a cold or the flu?

| YES |  |
| --- | --- |
| NO |  |

**IF YOU HAVE ANSWERED “NO” PLEASE SKIP TO QUESTION 15**

1. In the past 12 months, have you had a problem with sneezing, or a runny, or blocked nose when you DID NOT have a cold or the flu?

| YES |  |
| --- | --- |
| NO |  |

**IF YOU HAVE ANSWERED “NO” PLEASE SKIP TO QUESTION 15**

1. In the past 12 months, has this nose problem been accompanied by itchy-watery eyes?

| YES |  |
| --- | --- |
| NO |  |

1. In which of the past 12 months did this nose problem occur? (Please tick any which apply)

| January |  |
| --- | --- |
| February |  |
| March |  |
| April |  |
| May |  |
| June |  |

| July |  |
| --- | --- |
| August |  |
| September |  |
| October |  |
| November |  |
| December |  |

1. In the past 12 months, how much did this nose problem interfere with your daily activities?

| Not at all |  |
| --- | --- |
| A little |  |
| A moderate amount |  |
| A lot |  |

1. Have you ever had hay fever?

| YES |  |
| --- | --- |
| NO |  |

| YES |  |
| --- | --- |
| NO |  |

1. Was the hay fever diagnosed by a doctor or nurse?

**CORE QUESTIONNAIRE FOR ECZEMA**

1. Have you ever had an itchy rash that was coming and going for at least six months.

| Yes |
| --- |
| No |

**If you answered “NO” please skip to question 23**

18.Have you had this itchy rash at any time in the past 12 months.

| Yes |
| --- |
| No |

**If you answered “NO” please skip to Question 23**

19.Has this itchy rash at any time affected any of the places : the folds of the elbows , behind the knees, in front of the ankles, under the buttocks, or around the neck, ears, or eyes?

| Yes |
| --- |
| No |

20.At what age did this itchy rash first occur? under 2 years, age 2-4, or 5 or more years of age

|  |
| --- |

21.Has this itchy rash cleared completely at any time during the past 12 months

| Yes |
| --- |
| No |

22.In the past 12 months how often on average, have you been kept awake at night by this itchy rash?

| Never |
| --- |
| Less than one night |
| One or more nights per week |

23.Have you ever had eczema

| Yes |
| --- |
| No |

**General questionnaire**

1. How long have you lived in this SUBURB/ TOWNSHIP/SECTION?

| Less than 6 months |  |
| --- | --- |
| 6 to 12 months |  |
| 1 to 2 years |  |
| 1. years or longer |  |

1. How do you usually get to school?

| Walk |  |
| --- | --- |
| Taxi/Bus |  |
| Motor car |  |
| Combination |  |
| Other |  |

1. How far is the nearest Clinic or Hospital from your home?

| 15 minutes’ walk or 5-minute drive |  |
| --- | --- |
| 1 hour walk or 15-minute drive |  |
| more than an hour’s walk or more than  30-minute drive |  |

1. What type of house do you live in?

| Brick |  |
| --- | --- |
| Mud |  |
| Corrugated iron |  |
| Combination |  |
| Other |  |

1. How many rooms are in your house? (Excluding bathrooms)

|  |
| --- |

1. Do you have running water in the house?

| YES |  |
| --- | --- |
| NO |  |

1. In the past 12 months, how often, on average, did you eat or drink the following: (Please leave blank if you do not know what a food is)

| **Type of food** | **Never or occasionally** | **Once or twice per week** | **Three or more times per week** |
| --- | --- | --- | --- |
| Meat (e.g., beef, lamb, chicken, pork) |  |  |  |
| Seafood (including fish) |  |  |  |
| Fruit |  |  |  |
| Vegetables (green and root) |  |  |  |
| Pulses (peas, beans, lentils) |  |  |  |
| Cereal (including bread) |  |  |  |
| Pasta |  |  |  |
| Rice |  |  |  |
| Butter |  |  |  |
| Margarine |  |  |  |
| Nuts |  |  |  |
| Potatoes |  |  |  |
| Milk |  |  |  |
| Eggs |  |  |  |
| Fast food/burgers |  |  |  |

1. In the past 6 months, how often have you been absent from school?

| Never or occasionally |  |
| --- | --- |
| Once or twice per week |  |
| Three or more times a week |  |

1. During a normal week, how many hours a day (24hours) do you watch television?

| Less than 1 hour |  |
| --- | --- |
| 1 hour but less than 3 hours |  |
| 3 hours but less than 5 hours |  |
| 5 hours or more |  |

1. In your house, what fuel is usually used for cooking?

| Electricity |  |
| --- | --- |
| Gas |  |
| Paraffin |  |
| Open fires |  |
| Other – Please specify |  |

1. In your house, what fuel is usually used for heating?

| Electricity |  |
| --- | --- |
| Gas |  |
| Paraffin |  |
| Open fires (wood, coal) |  |
| Other – Please specify |  |

1. In the past 12 months, how often, on average, have you taken paracetamol (e.g., Panadol)?

| Never |  |
| --- | --- |
| At least once a year |  |
| At least once per month |  |

13. How many older brothers and sisters do you have?

| Brothers |  |
| --- | --- |
| Sisters |  |

14. How many younger brothers and brothers sisters do you have?

| Brothers |  |
| --- | --- |
| Sisters |  |

15. Were you born in this township/suburb?

| YES |  |
| --- | --- |
| NO |  |

16. How often do trucks pass through the street where you live, on weekdays?

| Never |  |
| --- | --- |
| Seldom |  |
| Frequently through the day |  |
| Almost all day |  |

17. Do you currently have a cat in your home?

| YES |  |
| --- | --- |
| NO |  |

18. In the past 12 months, have you had a cat in your home?

| YES |  |
| --- | --- |
| NO |  |

19. Do you currently have a dog in your home?

| YES |  |
| --- | --- |
| NO |  |

20. In the past 12 months, have you had a dog in your home?

| YES |  |
| --- | --- |
| NO |  |

21. Does your mother (or female guardian) smoke cigarettes?

| YES |  |
| --- | --- |
| NO |  |

22. Does your father (or male guardian) smoke cigarettes?

| YES |  |
| --- | --- |
| NO |  |

23. How many people living your house smoke cigarettes?

|  |
| --- |

People

24. In the past 30 days about how many days would you say you were in a place where someone smoked close to you (no complete physical barrier i.e., smoke got to you)?

|  | Never | 1-6 days | 7-10 days | 16-20 days | More than 20 days |
| --- | --- | --- | --- | --- | --- |
| At home |  |  |  |  |  |
| At school |  |  |  |  |  |
| In the car or transport |  |  |  |  |  |
| Restaurant |  |  |  |  |  |

**Air quality perceptions**

25. How would you rate the indoor air quality in your home?

| Good |  |
| --- | --- |
| Average |  |
| Poor |  |

26. How would you rate the outdoor air quality in your community?

| Good |  |
| --- | --- |
| Average |  |
| Poor |  |

27. **Please express how much do you agree or disagree on the following statements**

|  | **Strongly agree** | **Agree** | **Undecided** | **Disagree** | **Strongly disagree** |
| --- | --- | --- | --- | --- | --- |
| Improving environment is the responsibility of every citizen |  |  |  |  |  |
| Recycling programs should be put in place and promoted across the whole city |  |  |  |  |  |
| I am actively involved in cleaning up the environment |  |  |  |  |  |
| The pollution is out of my control, and I cannot do anything to change it |  |  |  |  |  |
| If I knew how to better contribute to a cleaner environment, I would act |  |  |  |  |  |
| I do not see the pollution as a health problem |  |  |  |  |  |

**THANK YOU!**
