# Supplementary material for: Association Between Self‐Reported Socio‐Economic Conditions, Air Pollution Sources and Eczema Symptoms Amongst Teenagers in Soshanguve, Tshwane Metropolitan Municipality, Gauteng Province, South Africa
Source: Public Health Chall. 2026 Jul 28;5(3):e70325. doi: 10.1002/puh2.70325 (PMC13411282; doi:10.1002/puh2.70325)
Supplement: Supplementary file 2 — Supporting information file 2: puh270325‐sup‐0002‐TableS1.docx [file PUH2-5-e70325-s002.docx]

**Supplementary material**

**Table S1: Prevalence of self-reported EE and current ES by sex among school children in Soshanguve.**

| **Outcome** | **Male** | **Female** | **Total** |
| --- | --- | --- | --- |
| **EE** | n (%) | n (%) | n (%) |
| Yes | 266 (19.4) | 379 (29.0) | 645 (24.0) |
| No | 1109 (80.7) | 929 (71.0) | 2038 (76.0) |
| Total | 1375 (100) | 1308 (100) | 2683 (100) |
| **Current ES** |  |  |  |
| Yes | 273 (19.4) | 475 (35.5) | 748 (27.2) |
| No | 1137 (80.6) | 865 (64.6) | 2002 (72.8) |
| Total | 1410 (100) | 1340 (100) | 2750 (100) |

n = number of participants.

% = percentage of participants.

Current ES = self-reported current eczema symptoms.

EE = self-reported ever having had eczema.

Note: Percentages were calculated using available (non-missing) responses for each outcome. Participants with missing, "don't know," or "not stated" responses were excluded using complete case (listwise deletion); therefore, the denominators vary across outcomes
